# Transcriptomics and Selection Pressure Analysis Reveals the Influence Mechanism of PLIN1 Protein on the Development of Small Size in Min Pigs

**DOI:** 10.3390/ijms24043947

**Published:** 2023-02-15

**Authors:** Qiao Liu, Liqun Yu, Ziwen Zhang, Yang Chang, Zhonghua Liu, Chunzhu Xu

**Affiliations:** 1 Key Laboratory of Animal Cellular and Genetics Engineering of Heilongjiang Province, College of Life Science, Northeast Agricultural University, Harbin 150030, China; 2 Key Laboratory of Molecular Medicine and Biotherapy, School of Life Sciences, Beijing Institute of Technology, Beijing 100081, China

**Keywords:** Min pig, body size variation, WGCNA, selection pressures, sacrificial culture

## Abstract

Body size is an important biological phenotypic trait that has attracted substantial attention. Small domestic pigs can serve as excellent animal models for biomedicine and also help meet sacrificial culture needs in human societies. Although the mechanisms underlying vertebral development regulating body size variation in domestic pigs during the embryonic period have been well described, few studies have examined the genetic basis of body size variation in post embryonic developmental stages. In this study, seven candidate genes—*PLIN1*, *LIPE*, *PNPLA1*, *SCD*, *FABP5*, *KRT10* and *IVL*—significantly associated with body size were identified in Min pigs, on the basis of weighted gene co-expression network analysis (WGCNA), and most of their functions were found to be associated with lipid deposition. Six candidate genes except for *IVL* were found to have been subjected to purifying selection. *PLIN1* had the lowest ω value (0.139) and showed heterogeneous selective pressure among domestic pig lineages with different body sizes (*p* < 0.05). These results suggested that *PLIN1* is an important genetic factor regulating lipid deposition and consequently affecting body size variation in pigs. The culture of whole pig sacrifice in Manchu during the Qing Dynasty in China might have contributed to the strong artificial domestication and selection of Hebao pigs.

## 1. Introduction

Domestic pigs (*Sus scrofa*) not only provide meat protein for humans [1] but also have been widely used in biomedical and comparative genomics research, owing to their physiological metabolic and genomic similarities to humans [2,3]. With the development of human society, the Chinese nation has developed a time-honored and rich culture of rearing domestic pigs, which have important roles in traditional sacrifice [4]. Long-term artificial selection and intensive breeding plans have led to considerable phenotypic diversity in body size of domestic pigs, thus making pigs an ideal model organism for studying animal body size variation, and the molecular mechanisms underlying body height and obesity in humans [5,6,7]. In recent years, the molecular regulatory mechanisms of body size or body weight in domestic pigs have been widely studied, and many genes and loci associated with body size regulation of domestic pigs have been identified [8,9,10,11]. Among them, *VRTN* and *IGF-1* are well studied and have been confirmed to be body size associated genes. *VRTN* encodes a transcription factor that regulates somite segmentation through the Notch signaling pathway during embryonic development, and controls the development, formation and number of thoracic vertebrae in mammals, thus influencing body size [12]. *IGF-1* affects body size by upregulating insulin sensitivity, prolonging bone and muscle growth stages, and increasing fat mass during mammalian postembryonic development [13,14]. Min pigs, a well-known breed in China and worldwide, have excellent meat quality [15] and strong cold resistance [16]. Ermin pigs (EM), which are large Min pigs, and Hebao pigs (HB), which are small Min pigs, have differentiated from their ancestors in terms of body size variation, thus providing an ideal model for exploring the mechanism of animal body size differences. The body length, height and weight are significantly greater in EM than HB, although the number of vertebrae differs by only one (Table S1 (Supplementary Materials)) [17]. Therefore, the body size difference between EM and HB is closely associated with the postembryonic growth and development stage.

With the continual development of high-throughput sequencing technology, RNA-seq has been widely used to study the transcriptional regulatory mechanisms of formation of important traits in domesticated animals, including cattle [18], pigs [19], chickens [20], and sheep [21]. The most representative method screens candidate genes closely associated with target traits through weighted gene co-expression network analysis (WGCNA) based on differentially expressed genes (DEGs) [22,23,24]. Wang et al. have conducted WGCNA on the transcriptome sequencing data of the longest dorsal muscle of Duroc pigs and identified potential candidate genes associated with intramuscular fat content in commercial pigs [25]. That work laid an important foundation for performing precise molecular breeding.

To further reveal the relationships between candidate genes and phenotypic traits, artificial or environmentally induced selection patterns in animals during domestication must be explored, because new phenotypic traits are selected and passed on to the next generation to increase a population’s prevalence during the domestication process [26]. After domestication is established, the relaxation of artificial or natural selection pressures—both environmental and anthropogenic—increases new mainly non-synonymous mutations, thus resulting in more phenotypic changes [27]. The identification of positively selected traits is therefore a valuable tool for identifying genes that may control important traits, thus enabling genetic variants to be associated with specific phenotypes [28]. Isolating any causal factors responsible for specific genetic differences is difficult, owing to the numerous selective pressures involved [29]. Therefore, the combination of WGCNA screening candidate genes and selection pressure analysis is an effective means of more accurate determination of associations between domestication genes and phenotypic traits.

In this study, EM and HB with the same genetic origin were used to screen candidate genes associated with body size variation. Transcriptome + functional enrichment + WGCNA + protein-protein interaction networks (PPI) were compared to estimate the intensity and pattern of selection pressure on the candidate genes in domestic pig populations with different body sizes, and to reveal the mechanisms and driving factors of the body size difference at the genetic level. The results may provide a new basis for targeted selection of new breeds of domestic pigs with different body types, because large body size has implications for meat use, and small body size has implications for medical use. Our findings also provide a theoretical basis for further investigation of human growth, obesity and muscle-associated diseases.

## 2. Results

### 2.1. Co-Expressed Gene Modules Associated with Body Size

**Figure 1:**
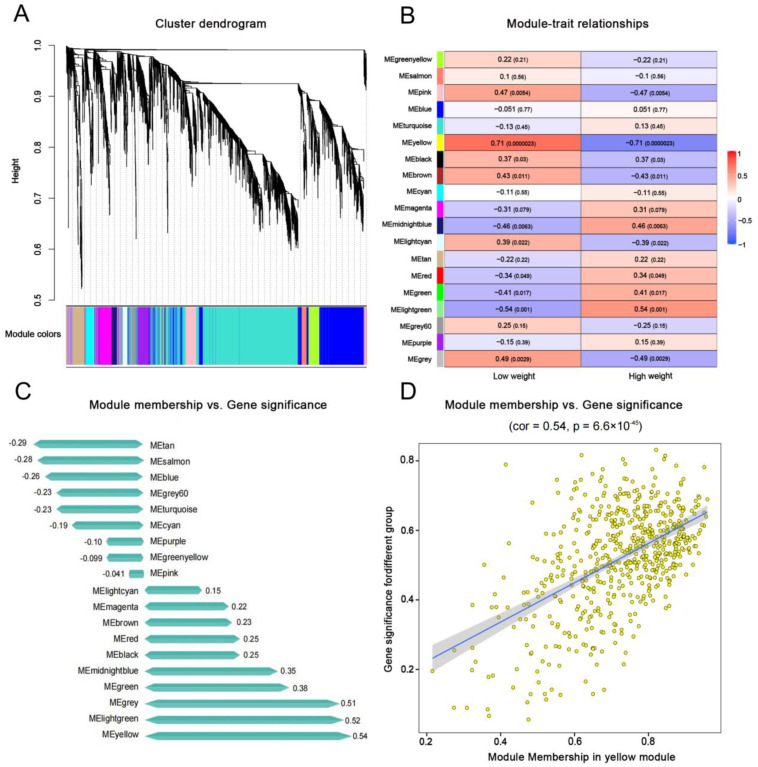
Co-expressed gene modules detected by weighted gene co-expression network analysis (WGCNA). (**A**) Cluster dendrogram showing the co-expression modules defined by WGCNA and labeled by colors. (**B**) Module-trait relationships showing the correlation between gene module and sample information, where the *x*-axis represents sample information, and the *y*-axis represents each gene module. In the panel, the darker the color, the higher the correlation, with red representing positive correlation and blue representing negative correlation. Significance values expressed as *p*-values are in parentheses. (**C**) Module membership and gene significance relationships of each module. (**D**) Scatterplot of Gene Significance (GS) for body type vs. Module Membership (MM) in yellow module.

After RNA quality control, sample HB18 was removed because of contamination. Cluster analysis was performed by calculating the correlation coefficient of gene expression levels of each sample, sample EM04 was excluded due to complete deviation from other samples. To construct a co-expression network, we applied WGCNA with FPKM values obtained from the RNA-Seq of 34 Min pigs. With an optimal power value of β = 7, R2 > 0.85, and the average connectivity tending to 0 (Figure S1), the power value was suitable for constructing a scale-free network. A total of 19 co-expression modules were identified on the basis of their similar expression patterns in Figure 1A. Among them, the turquoise module had the largest number of genes, at 1815, whereas the light green module had the smallest number of genes, at 56. Figure S2 shows the heat map of inter-module correlation. Only the yellow module, containing 576 genes, was identified among 19 modules (|r| > 0.60 and *p* < 0.05) to be significantly associated with the body size of Min pigs. This module was significantly and positively correlated with the small body size trait in HB (r = 0.71, *p* = 0.0000023) (Figure 1B). The correlation between GS and MM of each module was calculated, and the yellow module showed the highest correlation (0.54, *p* = 6.6 × 10^−45^) (Figure 1C,D), thus indicating that genes in the yellow module were most correlated with the body size of Min pigs.

### 2.2. Functional Enrichment Analysis of The Yellow Module

**Figure 2:**
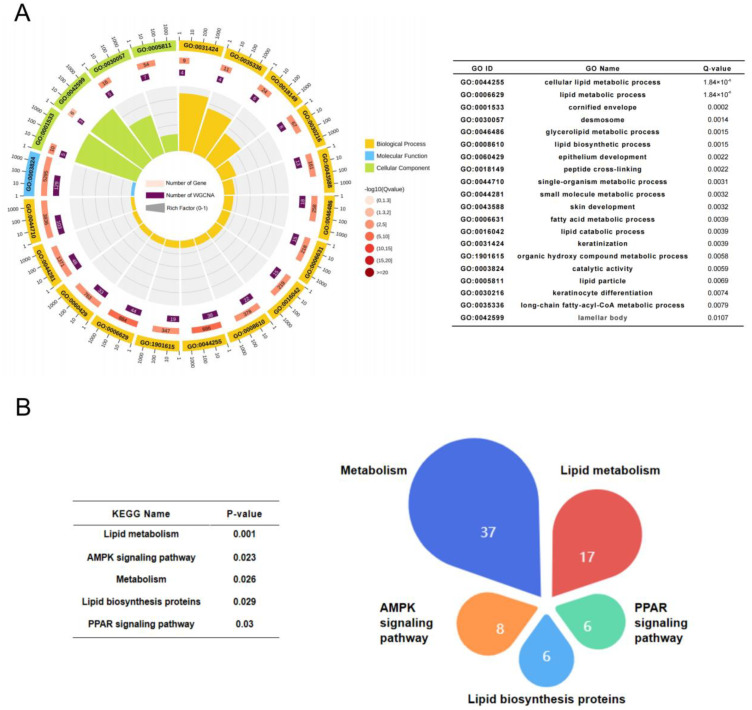
Co-expressed genes in yellow module. (**A**) The top 20 Gene Ontology (GO) terms identified with yellow module genes. (**B**) Pie chart of all significant pathways (Q-value < 0.05) in the yellow module. Each sector of the pie chart is proportional to the number of genes in the pathway.

To further understand the functions of genes, we classified the biological functions of 351 genes with GS and MM both >0.5 in the yellow module. We observed significant enrichment in 72 GO entries (Q-value < 0.05), as shown in Table S2. Among them, the top 20 enriched entries were all correlated with lipid deposition to varying degrees (Figure 2A). Five pathways were significantly enriched in KEGG terms with a Q-value < 0.05; these included lipid metabolism, metabolism, AMPK signaling pathway, lipid biosynthesis proteins and the PPAR signaling pathway. These pathways were all involved in lipid deposition processes, thus indicating that genes in the yellow module were closely associated with lipid deposition (Figure 2B).

### 2.3. Screening and Validation of Differentially Expressed Genes between EM and HB

**Figure 3:**
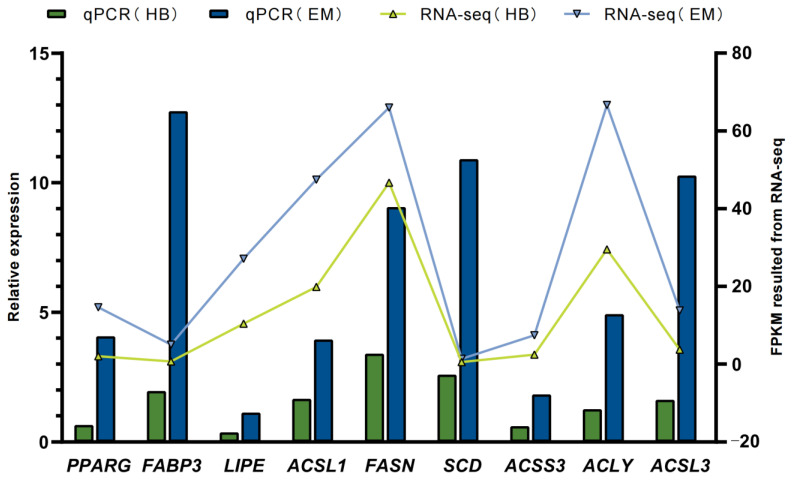
Validation of RNA-seq data by qRT-PCR. The line graph represents the FPKM value of RNA sequencing, and the bar graph represents the relative quantitative results of qRT-PCR.

A total of 707 DEGs were screened on the basis of FPKM values from the comparative transcriptome sequencing data of EM and HB. In HB, compared with EM, we observed 278 up-regulated genes and 429 down-regulated genes. Nine DEGs (*PPARG*, *FABP3*, *LIPE*, *ACSL1*, *FASN*, *SCD*, *ACSS3*, *ACLY* and *ACSL3*) were randomly selected to verify the transcriptome sequencing data by qRT-PCR, with the primers shown in Table S3. The expression trends in qRT-PCR and RNA-Seq data were similar, thus indicating the accuracy of the RNA-Seq data (Figure 3).

### 2.4. Protein-Protein Interaction Networks and Hub Gene Screening of Yellow Module Genes

**Figure 4:**
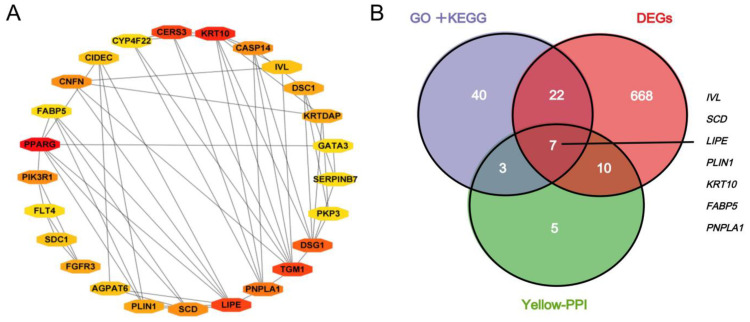
Hub genes in the yellow module. (**A**) Network visualization of the co-expression of 25 hub genes in yellow module. (**B**) The intersection in the Veen map is the most important candidate genes by various analysis methods.

A total of 576 genes in the yellow module were subjected to PPI network analysis, and 25 hub genes were identified with degree ≥ 5 (Figure 4A), among which 17 genes were differentially expressed between EM and HB (*p* < 0.01), with |log_2_foldchange| > 2. A total of 576 genes in the yellow module were analyzed for GO (Q < 0.001) and KEGG (Q < 0.05) enrichment, and 72 significantly enriched genes were identified, 29 of which were differentially expressed between EM and HB (*p* < 0.01), with |log_2_foldchange| > 2. A total of seven common genes were screened on the basis of joint GO-KEGG + PPI + DEGs analysis: *KRT10*, *LIPE*, *PNPLA1*, *SCD*, *PLIN1*, *IVL* and *FABP5* (Figure 4B). These genes were considered hub candidate genes associated with the body size difference between EM and HB.

### 2.5. Selection Pattern and Intensity Estimation of Body Size Candidate Genes in Domestic Pigs

Selection pressure analysis on seven candidate genes indicated that all six genes were subjected to purifying selection (0.139 < ω < 0.904) except for *IVL*, which was subjected to positive selection in all domestic pig populations. Among them, *PLIN1* had the lowest ω value (ω = 0.139), and *IVL* had the highest ω value (ω = 1.951), as shown in Table 1. Despite evidence that purifying selection acted on proteins overall, instances of statistically significant positive selection were observed across all loci of candidate genes in all pig breeds. A total of 152 codon positions were detected in seven candidate genes, among which 72 codons (48%) encoded the functional domains of proteins. The selection strengths varied among gene loci: *LIPE* (45) and *PNPLA1* (42) showed more codons under positive selection, whereas other genes, such as *FABP5*, had fewer positive selection sites (7).

The branch model was used to test differences in selection pressure among domestic pig populations with different body sizes. A comparison of the M0, M1 and M2 models indicated that the M1 model of *PLIN1* performed significantly better than the M0 model (*p* < 0.05). When small pigs were selected as the foreground branch, the M2 model also performed significantly better than the M0 model (*p* < 0.05), and the other six candidate genes were not significant, thus indicating heterogeneous selective pressure on *PLIN1* in different pig lineages.

## 3. Discussion

Because animal phenotypes are usually regulated by multiple genes, locating hub candidate genes with traditional unidimensional trait research methods is challenging. In this study, the WGCNA method was used to transform the association between thousands of genes and body size in EM and HB into an association between a multi-module gene and body size, thereby revealing the gene interactions without multiple hypothesis tests [30]. ML tests for detecting selection pressure have been widely accepted. The non-synonymous to synonymous substitution rate ratio (ω = dN/dS) provides a sensitive measure of selective pressure at the protein level, with ω values < 1, = 1, and > 1 indicating purifying selection, neutral evolution and diversifying selection, respectively [31]. The evolutionary process of genes can be well understood by selection pressure analysis. When positive selection genes were detected on the ancestral branch of cetaceans, their genes involved in osteoclast function underwent accelerated evolution, thus demonstrating that bone remodeling played an important role in their adaptation to the water environment [32]. Zhang et al. have analyzed the *TLR* genes of 102 amphibian species and found that purification selection played a major role in amphibian TLR evolution [33]. This study screened candidate genes associated with the body size variation in Min pigs through WGCNA, and analyzed candidate gene selection patterns and intensity in domestic pig populations with different body sizes. The underlying mechanism and factors driving body size variation in Min pigs were revealed in terms of both genetic variation and selection pressure, thus potentially enabling targeted selection and breeding of new pig varieties with different body sizes.

Body size variation in animals is a highly complex process affected by many factors. For some animal groups, human activities are the main force driving the change in body size [34]. Under the influence of artificial selection, abnormal skeletal system development in many animals leads to variations in body size. For example, the *IGF1* and *COL11A2* in purebred dog breeds have undergone strong artificial selection, and marked differences in bone size and shape eventually led to apparent differences in body height [35]. In addition, variation in the number of vertebrae is also an important factor leading to differences in animal body size. Similar cases have been reported in fish [36] and mammals [37]. The number of vertebrae and the body size of commercial pigs are significantly greater than those in local pigs. Studies have shown that *VRTN*, *NR6A1* and *IGF-1* are key genes that regulate the number of vertebrae in domestic pigs [9]. However, the number of vertebrae in two types of Min pigs differed by only one vertebra, but the body length, height and weight of EM were significantly higher than those of HB (Table S1), thus indicating that the difference in body size between EM and HB was not only associated with skeletal development, but also was closely associated with the stage of post embryonic growth and development. Through WGCNA combined with selection pressure analysis, this study indicated that *PLIN1* was closely associated with body size variation in Min pigs. On the basis of analysis of biological function, *PLIN1* belongs to the *PLIN* family, and periplasmic phospholipid protein is localized on the surfaces of lipid droplets in adipocytes and steroid-producing cells, which play central roles in lipid storage and catabolism [38]. The *PLIN1* gene has a regulatory role in diet-induced changes in body fat and energy metabolism in obese women [39] and therefore may be an important candidate gene associated with body size variation in Min pigs, by regulating lipid deposition. This gene might potentially be used as a gene target for breeding pigs with different body sizes in the future. Similarly, Huang et al. have found that 14 genes associated with body size variation in carnivores are strongly associated with obesity in humans, by increasing body size through augmenting body fat deposition [40]. Genes play key roles in producing body size variations in different populations, and screening for body size candidate genes may identify individuals with a predisposition to obesity and associated metabolic disease risks; provide new insights into the potential regulatory roles of genes in weight loss treatment; and further clarify the potential benefits of personalized medicine [41].

The domestication of animals has changed human lifestyles. After domestication, most domesticated animals have been further specifically bred by humans to produce various targeted phenotypes [42]. Domestic pigs were not only a source of daily protein, but also gradually became the most common sacrificial animal in China during the Neolithic period. Sacrifice represents humans’ inner aspirations and cultural preferences, and culture has had an important influence on animal domestication [4]. The origin of domestic pigs in northern China can be traced back to at least the early to middle Neolithic period 8000 years ago. Two complete pig skeletons were buried with humans at the Xinglongwa Site, thus implying that pigs had been endowed with special cultural connotations [43,44]. In the Hongshan culture, which is more than 5000 years old, people believed in shamans. During that period, livestock, most of which were pigs, were buried in the burial pits of beasts for sacrifice [45,46]. Archaeological evidence has confirmed that whole juvenile pigs were commonly used for sacrifice in the early Shang dynasty but were later replaced by adult pigs. Adult pigs needed to be cut into pieces as sacrifices because of their large size, thus reflecting the diverse forms of animal sacrifice in the Shang Dynasty, and emphasizing the absolute control and power of the ruling class [44]. In the development of domestic pigs in sacrificial culture, the selective domestication of black-furred domestic pigs by Chinese ancestors is a typical case of the inheritance of ancient sacrificial cultural practices [47]. During the Qing Dynasty, medium and large domestic pigs from northern China came to present-day northeastern China with the northward migration of the Han Chinese (Manchu-Han intermarriage). After more than 300 years of natural and artificial selection, the current breeds of EM and HB were formed [17]. The ancestors of the Manchu people in the Qing Dynasty were the inheritors of Hongshan culture. They preferred to sacrifice whole pigs to their ancestors, and choosing small adult pigs for sacrifice was considered more pious [46]. Thus, the sacrificial culture of Manchu was likely to have been a major cultural driving force in the artificially directed breeding of HB to obtain a small body size.

## 4. Materials and Methods

### 4.1. Animals and Sample Collection

A total of 36 dorsal muscle tissue samples were collected in this study, including 18 EM, denoted EM01 to EM18, from seven boars and 11 sows from the EM National Breeding Farm in Lanxi County, Heilongjiang Province, and 18 HB, denoted HB01 to HB18, from four boars and 14 sows from the HB National Breeding Farm in Lingyuan City, Liaoning Province. All individuals were collected in June 2019 and were healthy, randomly selected, 18-month-old adults. Samples were rapidly frozen in cryogenic liquid nitrogen and stored at an ultra-low temperature of −80 ℃ until further testing.

### 4.2. RNA Isolation and Sequencing

Total RNA was extracted from 36 muscle tissue samples with TRIzol reagent and then subjected to quality control. The RNA-seq library construction, Illumina sequencing and read mapping were performed at Personalbio (http://www.personalbio.cn/ (accessed on 23 April 2022)). For data analysis, paired reads were mapped to the *Sus scrofa* (11.1) genome assembly with HISAT2 [48] with default parameters. The unique mapped reads of each specific transcript were counted with HTSeq (v0.6.1) [49].

### 4.3. Weighted Gene Co-Expression Network Analysis

Modules with highly correlated genes were located with WGCNA, and the network was used to associate modules with external target traits to calculate module membership [50]. The WGCNA shiny plugin in TBtools (v1.098769) [51] was used to construct a gene co-expression network. Genes with FPKM values > 1 in more than 30 individuals were selected for a co-expression network setting, thus yielding 7000 genes. Outliers of sample clustering were removed before network construction. According to the scale-free network principle, when the correlation coefficient between log (k) of the number of connected nodes and log (pk) of the probability of node occurrence first reached 0.85, the corresponding weighting coefficient β was determined. The merge cut height was set to 0.25, the min module size was 50, and the generated cluster tree branches were defined as gene modules with the dynamic branch cutting tree method. Each branch represented a co-expression module, and genes with similar expression patterns within the same module were distinguished by different colors.

The module eigengene, which can be regarded as representative of the gene expression profiles of a module, is defined as the first principal component of a module of interest. To identify biologically meaningful modules and to select potential critical modules for downstream analysis, the WGCNA approach defines the module-trait relationships and gene significance (GS) of each module. Module-trait relationships >0.6 and *p*-value < 0.05 were used as the criteria for selecting target modules. Module membership (MM) represented the correlation between a single gene and the module in which it was located, and GS represented the correlation between individual genes of the module and the trait. If the correlation between GS and MM is significant, the central genes of the module are closely associated with the target trait [23], both of which can be used in further screening for hub candidate genes in the module. In the target module, genes with GS and MM both >0.5 were used for subsequent analysis.

### 4.4. Functional Enrichment Analysis of Co-Expression Modules

To explore the function of the target module screened by the co-expression network, we performed Gene Ontology (GO) and Kyoto Encyclopedia of Genes and Genomes (KEGG) enrichment analyses. The GO database describes genes on the basis of three aspects—molecular function, cellular component and biological process—to analyze the main biological functions performed by genes. The KEGG database was used to identify the main biochemical metabolic pathways or signal transduction pathways enriched in genes. In this study, GO Enrichment and KEGG Enrichment Analyze in TBtools were used to analyze genes with GS and MM both >0.5 in target modules, and a Q-value < 0.05 was used as the threshold. Visualization was performed with the Kidio online platform (https://www.omicshare.com (accessed on 15 July 2022)) cloud tool.

### 4.5. Differential Expression Analysis and Real-Time Quantitative Polymerase Chain Reaction Analysis

The DEGseq [52] software package was used to analyze the differential expression of genes on the basis of fragments per kilobase of exon model per million mapped fragments (FPKM) values, and DEGs were screened with fold-change and significance tests. Under the condition of *p* < 0.01 and |log_2_foldchange| > 2, genes with significant differences were screened for subsequent analysis. The transcriptome data were further validated with real-time quantitative polymerase chain reaction (qRT-PCR) analysis. The gene primers were designed in Primer Premier v5.0. [53] software, and the relative expression levels of nine randomly selected genes were analyzed. Three biological replicates of each tissue sample, and at least three technical replicates of each biological replicate were used in the subsequent expression analysis. The transcript level of each gene was normalized to that of *PPIA* for each sample, and fold changes in relative gene expression were calculated with the 2^−∆∆CT^ method.

### 4.6. Protein-Protein Interaction Network of Modules and Candidate Genes Screening for Body Size

Interactions among proteins are involved in biological signal transmission, gene expression regulation, energy and material metabolism, cell cycle regulation and other life processes. Therefore, understanding the working principles of proteins in biological systems and the functional relationships among proteins by systematically analyzing the interaction relationships of multiple proteins in biological systems is important. The existing protein interactions in the STRING database (https://cn.string-db.org (accessed on 4 August 2022)) were used to construct a molecular network associated with the body size of Min pigs. On the basis of the connection relationships between protein nodes output from the STRING database, we used Cytoscape (v3.9.1) [54] software to draw the network relationship map and the Degree topology algorithm in the cytoHubba plugin tool to predict the key nodes in the gene network associated with body size. The central gene sets with close interaction in the PPI network were identified on the basis of Degree calculation results exceeding a value of five. The core sub-network in the global network was also determined. To identify the main candidate genes associated with body size, the screening criteria for GO entries enriched in the target module were set accordingly. The DEGs enriched in GO terms with Q < 0.001 were intersected with DEGs enriched in KEGG pathway terms with Q < 0.05; the DEGs in the union were then intersected with DEGs in the PPI core sub-network.

### 4.7. Acquisition of Candidate Gene Sequences Associated with Body Size in Domestic Pigs

In this study, the selected candidate genes associated with body size were analyzed among 14 pig breeds: HB, EM, Wuzhishan pigs, Tibetan pigs, Jinhua pigs, Meishan pigs, Bamei pigs, Rongchang pigs, Duroc pigs, Landrace pigs, Large white pigs, Berkshire pigs, Pietrain pigs and Hampshire pigs. Gene sequences of HB and EM were obtained from the transcriptome sequencing. Genomes and annotation files of 12 other pig breeds were downloaded from the Ensembl database (http://www.ensembl.org (accessed on 21 August 2022)) as shown in Table S4, and their candidate gene sequences were obtained with BLAST in TBtools (E-value < 1 × 10^−5^) with Duroc pigs as a reference. The MUSCLE program in MEGA11 [55] was used for multiple sequence alignment and adjustment.

### 4.8. Analysis of Selection Patterns of Candidate Genes Associated with Body Size in Domestic Pigs

To detect the selection pressure on candidate genes of domestic pigs with different body sizes, we grouped the 14 pig breeds by body size. EM, Jinhua pigs, Meishan pigs, Baimei pigs, Rongchang pigs, Duroc pigs, Large white pigs, Landrace pigs, Berkshire pigs, Pietrain pigs and Hampshire pigs were classified as large domestic pigs. HB, Wuzhishan pigs and Tibetan pigs were classified as small domestic pigs. The phylogenetic tree of candidate genes in domestic pigs was constructed with the maximum likelihood (ML) method, and selection pressure analysis was performed with EasyCodeML1.4 [56]. The positive selection hypothesis of candidate genes was evaluated on the basis of the site model, which allows different amino acid sites to be subjected to variable selection pressures while different branches of the phylogenetic tree are subjected to the same selection pressure. Evidence of positive selection at individual codons of each body size candidate gene was inferred with the two nested models by comparison of M7 with M8 in the site model. Likelihood ratio tests (LRTs) were subsequently performed. The Bayes Empirical Bayes approach was used to determine site-specific posterior probabilities of positive selection exceeding 99% in cases in which the LRT was significant. The mixed effect model and fixed effect model (glycoprotein FEL) from the DataMonkey website [57] (http://www.datamonkey.org/analyses (accessed on 9 October 2022)) were used to validate the identified sites. Sites with β+ > α and *p* < 0.1 (by LRT) were identified as positive selection sites. The heterogeneity in ω between large and small domestic pigs was investigated by comparison of the one-ratio model (M0), the free-ratio model (M1) and the two-ratio model (M2) with the LRT method. In these models, different ω values were assigned for domestic pig groups with different body sizes: in M0, all lineages had a single ω ratio, M1 allowed all branches to have different ω values, and M2 had different ω values in the foreground and background branches.

## 5. Conclusions

In this study, seven candidate genes—*PLIN1, LIPE, PNPLA1, SCD, FABP5, KRT10* and *IVL*—significantly associated with body size were identified in Min pigs, and most of their functions were found to be associated with lipid deposition. In addition, our findings unraveled that the *PLIN1* gene may be an important candidate gene associated with body size variation in Min pigs, and the sacrificial culture of Manchu was likely to have been a major cultural driving force in the artificially directed breeding of HB to obtain a small body size.

## Figures and Tables

**Table 1:** Positive selection at amino acid sites of seven candidate genes in domestic pigs.

Gene	No. of Sequences	−2ΔlnL	PAML	FEL (*p* < 0.1)	MEME (*p* < 0.1)	All Sites	Functional Domain Sites	ω
*KRT10*	20	44.11	23, 25, 26, 27, 28	246	251, 464, 252, 486, 249, 500, 499, 24, 495, 501, 277, 484, 276, 320, 345	21	18	0.904
*LIPE*	39	106.64	83, 454, 461, 467, 558, 631, 632, 640, 641, 642, 653, 654	**194**, 38	22, **194**, 563, 148, 669, 736, 741, 742, 788, 562, 565, 567, 576, 744, 752, 773, 790, 743, 746, 756, 570, 738, 785, 733, 753, 772, 759, 38, 740, 11, 568, 578	45	0	0.575
*PNPLA1*	17	356.87	**423**, **424**, **430**, **432**, **435**, 437, 442, 446, **447**, 448, 449, **450**, 451, 452, **453**, 454, **455**, **457**, 458, 459, **460**, 461, 462, 463, 464, 465, 466, 467, 468, 469, 470, 471, 472, 473, 475, 476, 477, 478	None	294, **423**, **424**, **430**, **432**, **435**, **447**, **450**, **453**, **455**, 456, **457**, **460**, 487, 490	42	37	0.687
*SCD*	33	33.99	108, 109, 111, 124, 287, 326, 330	None	170, 424, 380, 387, 405, 386	13	1	0.569
*PLIN1*	30	16.29 *	5, **11**	None	8, **11**, 208, 14, 408, 429, 428, 426, 481	10	6	0.139
*IVL*	15	20.84	61, 93	**150**	205, 382, 204, 207, 154, 380, 217, 337, 377, 202, 358, **150**	14	10	1.951
*FABP5*	24	13.82	5	None	9, 221, 213, 212, 215, 211	7	0	0.668

Note: Positively selected sites inferred by both methods are underlined and amino acid positions are named based on the seven candidate genes in *Sus scrofa*. * represents *p*-value < 0.05.

## Data Availability

The data presented in this study are available on request from the corresponding author without any restrictions.

## Supplementary Materials

The supporting information can be downloaded at: https://www.mdpi.com/article/10.3390/ijms24043947/s1.
