# Supplementary material for: Transcriptomics and Selection Pressure Analysis Reveals the Influence Mechanism of PLIN1 Protein on the Development of Small Size in Min Pigs
Source: Int J Mol Sci. 2023 Feb 15;24(4):3947. doi: 10.3390/ijms24043947 (PMC9960057; doi:10.3390/ijms24043947)
Supplement: Supplementary file 1 [file ijms-24-03947-s001.zip › ijms-2189766-supplementary.pdf]

Table S1 Body measurement data of Ermin pig and Hebao pig (wang et al., 2011)

Table S2 Gene ontology enrichment analysis for genes in the yellow module

Table S3 Primers used for the qRT-PCR validation

Table S4 Summary of identified pig transcripts

Figure S1 Determination of soft threshold.

Figure S2 Heat map of inter-module correlation

Table S1 Body measurement data of Ermin pig and Hebao pig (wang et al., 2011)

Breed	Sex	Body Weight（kg）	Body length（cm）	Body height（cm）	Circumference（cm）	Spine number
Ermin pig	Male	227.10±8.70	152.20±0.81	89.10±0.71	147.30±0.89	35
	Female	181.40±10.27	147.20±1.26	84.00±0.42	137.90±1.25	35
Hebao pig	Male	93.30±0.76	124.50±0.47	63.50±0.72	105.25±0.96	34
	Female	81.80±0.64	106.75±0.60	59.60±0.60	97.00±0.98	34

Table S2 Gene ontology enrichment analysis for genes in the yellow module

Class	ID	Descrption	Gene number	Q-value
Biological Process	GO:0044255	cellular lipid metabolic process	38	1.84E-06
Biological Process	GO:0006629	lipid metabolic process	44	1.84E-06
Cellular Component	GO:0001533	cornified envelope	5	0.000175376
Cellular Component	GO:0030057	desmosome	5	0.001386936
Biological Process	GO:0046486	glycerolipid metabolic process	18	0.001476409
Biological Process	GO:0008610	lipid biosynthetic process	22	0.00154499
Biological Process	GO:0060429	epithelium development	33	0.002163421
Biological Process	GO:0018149	peptide cross-linking	6	0.002163421
Biological Process	GO:0044710	single-organism metabolic process	103	0.003082204
Biological Process	GO:0044281	small molecule metabolic process	48	0.003209151
Biological Process	GO:0043588	skin development	13	0.003209151
Biological Process	GO:0006631	fatty acid metabolic process	15	0.003879192
Biological Process	GO:0016042	lipid catabolic process	15	0.003879192
Biological Process	GO:0031424	keratinization	4	0.003879192
Biological Process	GO:1901615	organic hydroxy compound metabolic process	19	0.005827007
Molecular Function	GO:0003824	catalytic activity	129	0.005930316
Cellular Component	GO:0005811	lipid particle	7	0.006958703
Biological Process	GO:0030216	keratinocyte differentiation	8	0.007373908
Biological Process	GO:0035336	long-chain fatty-acyl-CoA metabolic process	4	0.007897133
Cellular Component	GO:0042599	lamellar body	38	0.010736356
Biological Process	GO:0044242	cellular lipid catabolic process	3	0.011658041
Biological Process	GO:0030855	epithelial cell differentiation	11	0.011658041
Biological Process	GO:0006650	glycerophospholipid metabolic process	20	0.011658041
Biological Process	GO:0048662	negative regulation of smooth muscle cell proliferation	13	0.011658041

Biological Process	GO:0006066	alcohol metabolic process	5	0.011658041
Biological Process	GO:0048513	animal organ development	15	0.017758895
Biological Process	GO:0035337	fatty-acyl-CoA metabolic process	62	0.019887505
Biological Process	GO:0006641	triglyceride metabolic process	4	0.019887505
Biological Process	GO:0008544	epidermis development	7	0.019887505
Biological Process	GO:0019637	organophosphate metabolic process	12	0.019887505
Biological Process	GO:0043542	endothelial cell migration	29	0.019999461
Biological Process	GO:0006633	fatty acid biosynthetic process	10	0.021282736
Biological Process	GO:0007166	cell surface receptor signaling pathway	8	0.022436663
Biological Process	GO:0032868	response to insulin	55	0.022976037
Biological Process	GO:0052547	regulation of peptidase activity	10	0.023409838
Molecular Function	GO:0016746	transferase activity, transferring acyl groups	16	0.025689688
Biological Process	GO:0009913	epidermal cell differentiation	14	0.026191886
Biological Process	GO:0010466	negative regulation of peptidase activity	9	0.026308242
Biological Process	GO:0044283	small molecule biosynthetic process	12	0.026308242
Biological Process	GO:0032869	cellular response to insulin stimulus	18	0.028186796
Biological Process	GO:0070887	cellular response to chemical stimulus	9	0.028186796
Biological Process	GO:0009888	tissue development	54	0.028186796
Molecular Function	GO:0033293	monocarboxylic acid binding	41	0.028504086
Biological Process	GO:0019216	regulation of lipid metabolic process	6	0.029834741
Biological Process	GO:0006663	platelet activating factor biosynthetic process	12	0.029834741
Biological Process	GO:0035962	response to interleukin-13	2	0.029834741
Biological Process	GO:0035963	cellular response to interleukin-13	2	0.029834741
Biological Process	GO:0046469	platelet activating factor metabolic process	2	0.029834741
Biological Process	GO:0043434	response to peptide hormone	2	0.031428207
Biological Process	GO:0032787	monocarboxylic acid metabolic process	11	0.031428207

Biological Process	GO:0042127	regulation of cell proliferation	17	0.031428207
Biological Process	GO:0071310	cellular response to organic substance	33	0.031428207
Biological Process	GO:0006636	unsaturated fatty acid biosynthetic process	46	0.031428207
Biological Process	GO:0050891	multicellular organismal water homeostasis	5	0.031428207
Biological Process	GO:0033561	regulation of water loss via skin	5	0.031842257
Biological Process	GO:0046503	glycerolipid catabolic process	4	0.033880984
Biological Process	GO:0010565	regulation of cellular ketone metabolic process	5	0.033880984
Biological Process	GO:0006665	sphingolipid metabolic process	7	0.034474693
Molecular Function	GO:0016755	transferase activity, transferring amino-acyl groups	8	0.03463752
Molecular Function	GO:0016289	CoA hydrolase activity	4	0.037550739
Molecular Function	GO:0042015	interleukin-20 binding	4	0.037550739
Biological Process	GO:0006639	acylglycerol metabolic process	2	0.038174931
Biological Process	GO:0045017	glycerolipid biosynthetic process	7	0.039719003
Biological Process	GO:0006638	neutral lipid metabolic process	8	0.039719003
Biological Process	GO:0030104	water homeostasis	7	0.039721332
Biological Process	GO:0010875	positive regulation of cholesterol efflux	5	0.042158639
Biological Process	GO:0006644	phospholipid metabolic process	3	0.042158639
Biological Process	GO:1901565	organonitrogen compound catabolic process	14	0.042158639
Biological Process	GO:0006637	acyl-CoA metabolic process	12	0.046533706
Biological Process	GO:0035383	thioester metabolic process	6	0.046533706
Cellular Component	GO:0005640	nuclear outer membrane	6	0.04876628
Molecular Function	GO:0030414	peptidase inhibitor activity	3	0.049114099

Table S3 Primers used for the qRT-PCR validation

Gene	Upstream primer	Downstream primer	Product length(bp)
FABP3	CTGGGAGTGGAGTTTGATGAGAC	CCATGGGTGAGTGTCAGGAT	164
LIPE	CGCACAATGACACAGTCACTGGT	AGGCAGCGGCCGTAGAAGCA	497
FASN	GCTTGTCCTGGGAAGAGTGTA	AGGAACTCGGACATAGCGG	115
SCD	AAGGAACTAGAAGGCTGCTC	TGTAGAGCAGCAGCCATCAC	211
PPARG	CACTCCACACTATGAAGACA	ACAGGCTCCACTTTGATG	111
ACSL1	CTCATAGCGATTGTGGTT	CAGTCTCAGCATGTCTTC	135
ACSL3	ATTGGCTGTCCTATGAAGAAGT	CATTCTGCTCTGGTCTCACA	118
ACSS3	AGAACGATGTGACGTGGAG	GAGGAGGCGTTTTGGAGTT	140
ACLY	CCTTATCCTGAATGTAGACGGTTT	AATGAAGCCCATACTCCTTCC	151

Table S4 Summary of identified pig transcripts

Gene	Taxa	Latin name	Breed	Ensembl ID or TRINITY ID
LIPE	Big	*Sus scrofa*	Ermin	TRINITY_DN3808_c0_g1_i1
		Sus scrofa	Jinhua	ENSSSCT00060099954,ENSSSCT00060099783,ENSSSCT00060099844,ENSSSCT00060100058
		Sus scrofa	Bamei	ENSSSCT00050063750,ENSSSCT00050063732,ENSSSCT00050063741
		Sus scrofa	Duroc	ENSSSCT00000058198,ENSSSCT00000003352
		Sus scrofa	Largewhite	ENSSSCT00025010621,ENSSSCT00025010592,ENSSSCT00025010605,ENSSSCT00025010641
		Sus scrofa	Meishan	ENSSSCT00040045965,ENSSSCT00040045859,ENSSSCT00040045913,ENSSSCT00040046012
		Sus scrofa	Hampshire	ENSSSCT00035058557,ENSSSCT00035058527,ENSSSCT00035058544,ENSSSCT00035058564
		Sus scrofa	Barkshire	ENSSSCT00065027793,ENSSSCT00065027763,ENSSSCT00065027753,ENSSSCT00065027770
		Sus scrofa	Pietrain	ENSSSCT00055015443,ENSSSCT00055015366
		Sus scrofa	Rongchang	ENSSSCT00030028053,ENSSSCT00030028032,ENSSSCT00030028012
		Sus scrofa	Landrace	ENSSSCT00045058234,ENSSSCT00045058147,ENSSSCT00045058302
	Small	*Sus scrofa*	Hebao	TRINITY_DN10856_c0_g1_i1
		Sus scrofa	Tibetan	ENSSSCT00015070418,ENSSSCT00015070241,ENSSSCT00015070019
		Sus scrofa	Wuzhishan	ENSSSCT00005031110
PLIN1	Big	*Sus scrofa*	Ermin	TRINITY_DN2410_c0_g3_i2
		Sus scrofa	Jinhua	ENSSSCT00060100242,ENSSSCT00050034243,ENSSSCT00050034249
		Sus scrofa	Bamei	ENSSSCT00050034236
		Sus scrofa	Duroc	ENSSSCT00000002067,ENSSSCT00000002066
		Sus scrofa	Largewhite	ENSSSCT00025105825,ENSSSCT00025105841,ENSSSCT00025105850
		Sus scrofa	Meishan	ENSSSCT00040101023,ENSSSCT00040101067
		Sus scrofa	Hampshire	ENSSSCT00035076116,ENSSSCT00035076125,ENSSSCT00035076132
		Sus scrofa	Barkshire	ENSSSCT00065059885,ENSSSCT00065059895,ENSSSCT00065059891
		Sus scrofa	Pietrain	ENSSSCT00055032144,ENSSSCT00055032243

		Sus scrofa	Rongchang	ENSSSCT00030034415,ENSSSCT00030034420
		Sus scrofa	Landrace	ENSSSCT00045047800,ENSSSCT00045047928,ENSSSCT00045048018
		Sus scrofa	Hebao	TRINITY_DN117_c1_g1_i1
	Small	*Sus scrofa*	Tibetan	ENSSSCT00015033266,ENSSSCT00015033341
		Sus scrofa	Wuzhishan	ENSSSCT00005040738,ENSSSCT00005040777
		Sus scrofa	Ermin	TRINITY_DN660_c0_g1_i26,TRINITY_DN660_c0_g1_i32
		Sus scrofa	Jinhua	ENSSSCT00060012558
		Sus scrofa	Bamei	ENSSSCT00050055551
		Sus scrofa	Duroc	ENSSSCT00000028214
		Sus scrofa	Largewhite	ENSSSCT00025057168
	Big	*Sus scrofa*	Meishan	ENSSSCT00040005962,ENSSSCT00040005967
		Sus scrofa	Hampshire	ENSSSCT00035053061
KRT10		*Sus scrofa*	Barkshire	ENSSSCT00065003363
		Sus scrofa	Pietrain	ENSSSCT00055050718
		Sus scrofa	Rongchang	ENSSSCT00030043492,ENSSSCT00030043505
		Sus scrofa	Landrace	ENSSSCT00045014925,ENSSSCT00045014872
		Sus scrofa	Hebao	TRINITY_DN4410_c0_g1_i7
	Small	*Sus scrofa*	Tibetan	ENSSSCT00015060935
		Sus scrofa	Wuzhishan	ENSSSCT00005049067
		Sus scrofa	Ermin	TRINITY_DN989_c1_g1_i1,TRINITY_DN989_c1_g1_i5
		Sus scrofa	Jinhua	ENSSSCT00060108051
		Sus scrofa	Bamei	ENSSSCT00050075236
PNPLA1	Big	*Sus scrofa*	Duroc	ENSSSCT00000093198,ENSSSCT00000001738
		Sus scrofa	Largewhite	ENSSSCT00025009259
		Sus scrofa	Meishan	ENSSSCT00040010269
		Sus scrofa	Hampshire	ENSSSCT00035013219

		Sus scrofa	Barkshire	ENSSSCT00065045787
		Sus scrofa	Pietrain	ENSSSCT00055021313
		Sus scrofa	Rongchang	ENSSSCT00030101583,ENSSSCT00030101552
		Sus scrofa	Landrace	ENSSSCT00045051875
		Sus scrofa	Hebao	TRINITY_DN4410_c0_g1_i7
	Small	*Sus scrofa*	Tibetan	ENSSSCT00015071165
		Sus scrofa	Wuzhishan	ENSSSCT00005047873
		Sus scrofa	Ermin	TRINITY_DN4_c0_g1_i4
		Sus scrofa	Jinhua	ENSSSCT00060087410,ENSSSCT00060087395
		Sus scrofa	Bamei	ENSSSCT00050105969,ENSSSCT00050105951,ENSSSCT00050105956
		Sus scrofa	Duroc	ENSSSCT00000074035
		Sus scrofa	Largewhite	ENSSSCT00025077027,ENSSSCT00025076897
	Big	*Sus scrofa*	Meishan	ENSSSCT00040032128,ENSSSCT00040032165,ENSSSCT00040032079
		Sus scrofa	Hampshire	ENSSSCT00035046939,ENSSSCT00035046926,ENSSSCT00035046934
SCD		*Sus scrofa*	Barkshire	ENSSSCT00065076049,ENSSSCT00065076040,ENSSSCT00065076045
		Sus scrofa	Pietrain	ENSSSCT00055005503,ENSSSCT00055005482,ENSSSCT00055005489
		Sus scrofa	Rongchang	ENSSSCT00030094149,ENSSSCT00030094183,ENSSSCT00030094112
		Sus scrofa	Landrace	ENSSSCT00045024773,ENSSSCT00045024728,ENSSSCT00045024757
		Sus scrofa	Hebao	TRINITY_DN14965_c0_g1_i1
	Small	*Sus scrofa*	Tibetan	ENSSSCT00015091855
		Sus scrofa	Wuzhishan	ENSSSCT00005045161,ENSSSCT00005045104,ENSSSCT00005045125
		Sus scrofa	Ermin	TRINITY_DN4012_c3_g1_i2,TRINITY_DN4012_c3_g1_i1
		Sus scrofa	Jinhua	ENSSSCT00060067622
FABP5	Big	*Sus scrofa*	Bamei	ENSSSCT00050074711,ENSSSCT00050074717
		Sus scrofa	Duroc	ENSSSCT00000006747,ENSSSCT00000054753
		Sus scrofa	Largewhite	ENSSSCT00025078212,ENSSSCT00025078334

		Sus scrofa	Meishan	ENSSSCT00040078336
		Sus scrofa	Hampshire	ENSSSCT00035029459,ENSSSCT00035029468
		Sus scrofa	Barkshire	ENSSSCT00065036041,ENSSSCT00065036047
		Sus scrofa	Pietrain	ENSSSCT00055018290,ENSSSCT00055018258
		Sus scrofa	Rongchang	ENSSSCT00030059372,ENSSSCT00030059354
		Sus scrofa	Landrace	ENSSSCT00045012791,ENSSSCT00045012750
		Sus scrofa	Hebao	TRINITY_DN1794_c5_g1_i1,TRINITY_DN1794_c5_g1_i2
	Small	*Sus scrofa*	Tibetan	ENSSSCT00015035652
		Sus scrofa	Wuzhishan	ENSSSCT00005026127
		Sus scrofa	Ermin	TRINITY_DN2334_c0_g1_i7,TRINITY_DN2334_c0_g1_i3
		Sus scrofa	Jinhua	ENSSSCT00060024020
		Sus scrofa	Bamei	ENSSSCT00050049597,ENSSSCT00050049604
		Sus scrofa	Duroc	ENSSSCT00000007226
		Sus scrofa	Largewhite	ENSSSCT00025080485
	Big	*Sus scrofa*	Meishan	-
		Sus scrofa	Hampshire	ENSSSCT00035004873
IVL		*Sus scrofa*	Barkshire	ENSSSCT00065045213
		Sus scrofa	Pietrain	ENSSSCT00055012667
		Sus scrofa	Rongchang	-
		Sus scrofa	Landrace	ENSSSCT00045061697
		Sus scrofa	Hebao	TRINITY_DN1877_c0_g1_i4,TRINITY_DN1877_c0_g1_i2
	Small	*Sus scrofa*	Tibetan	ENSSSCT00015081463
		Sus scrofa	Wuzhishan	ENSSSCT00005020065

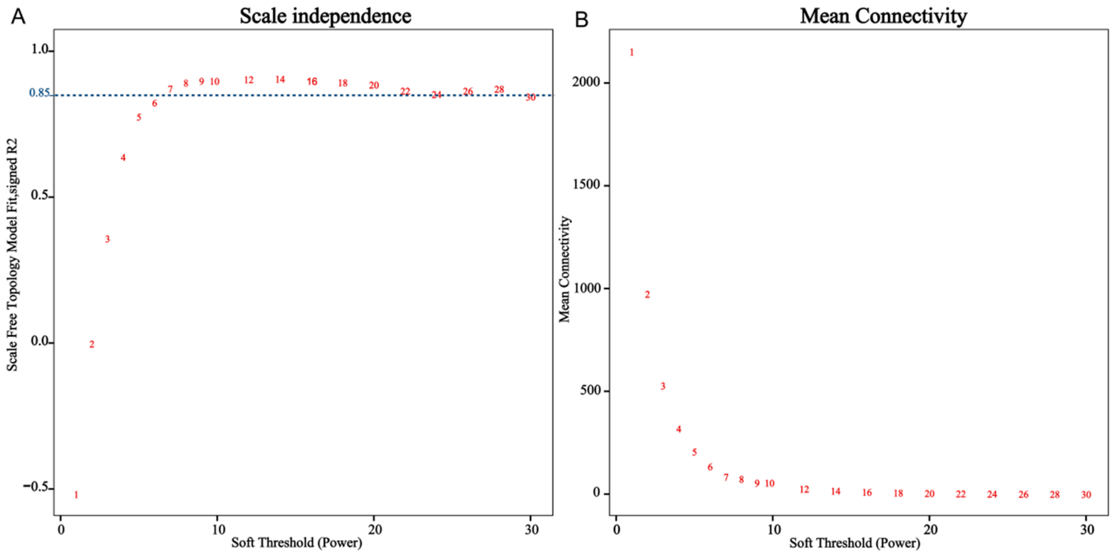

Figure S1 Determination of soft threshold. The abscissa represents the soft threshold (β). (A)Ordinate corresponds to the index of scale free network model. (B)The average link degree of each soft threshold.

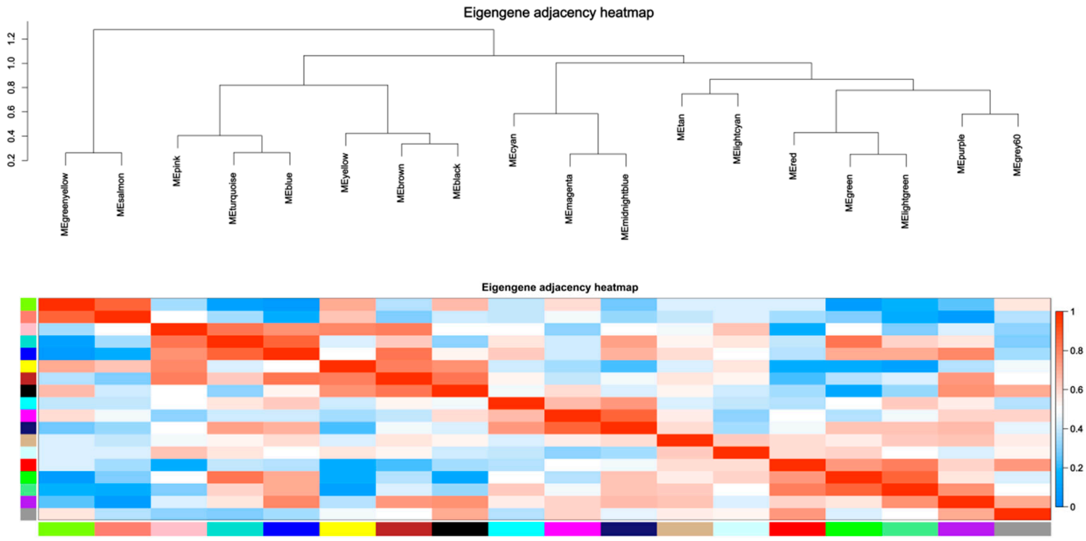

Figure S2 Heat map of inter-module correlation.
